# Netrin‐1 regulates ERK1/2 signaling pathway and autophagy activation in wear particle‐induced osteoclastogenesis

**DOI:** 10.1002/cbin.11544

**Published:** 2021-01-13

**Authors:** Lei Wang, Zhibiao Gao, Jie Zhang, Yulong Huo, Qiang Xu, Yusheng Qiu

**Affiliations:** ^1^ Department of Orthopedics The First Affiliated Hospital of Xi'an Jiaotong University Xi'an Shaanxi China; ^2^ Department of Orthopedics Affiliated Hospital of Yan'an University Yan'an Shaanxi China

**Keywords:** abnormal osteoclastogenesis, ERK1/2 signaling pathway, Netrin‐1, RAW 264.7 cell morphology, the air pouch model of bone resorption, titanium (Ti) wear particles

## Abstract

**Background:**

Artificial joint replacement surgery is often accompanied by osteolysis induced aseptic loosening around the prosthesis. Wear particles from joint replacement are thought to be one of the main factors leading to local inflammation and osteolysis at the prosthesis site. The aim of this study was to investigate the molecular mechanism of osteoclast formation and dissolution induced by wear particles and the potential roles of Netrin‐1, the ERK1/2 pathway and autophagy activation in this process.

**Methods:**

The messenger RNA levels in cells and tissues were detected with real‐time quantitative PCR. The western blotting was used to detect the expression of proteins. A CCK‐8 kit was used to detect the viability of RAW 264.7 cells. Moreover, an air pouch model of bone resorption was established. Immunohistochemistry was used to detect the expression of TRAP and Netrin‐1 in rat bone tissue. Cell culture supernatants were collected in the rat air pouch model of bone resorption, and the levels of RANKL and OPG were detected with enzyme‐linked immunosorbent assay. The protein levels of TRAP and Netrin‐1 in bone tissue were examined by immunohistochemistry.

**Results:**

Titanium wear particles induced osteoclast formation and autophagy activation. Moreover, blocking autophagy suppressed the osteoclastogenesis after exposure to wear particles in vitro. The activation of the ERK1/2 pathway and the overexpression of Netrin‐1 were both found to play important roles in osteoclastogenesis mediated by autophagy. Moreover, 3‐MA effectively decreased the secretion of proinflammatory cytokines mediated by wear particles.

**Conclusion:**

Blockade of autophagy inhibits the osteoclastogenesis and inflammation induced by wear particles, thus potentially providing novel treatment strategies for abnormal osteoclastogenesis and aseptic prosthesis loosening induced by wear particles.

**Abbreviations**
ATG: autophagy‐related
MA: 3‐methyladenine
MAPK: mitogen‐activated protein kinase
MOI: multiplicity of infection
NF‐κB: nuclear factor‐κB
PFA: paraformaldehyde
RANK: receptor activator of nuclear factor‐κB
RIPA: radioimmune precipitation assay

## 1 INTRODUCTION

As an important treatment for arthropathy, artificial joint replacement surgery is used to relieve joint pain and restore joint function, by using materials such as metal, polymeric polyethylene, or ceramic (Bouhidel et al., 2015; Bozic et al., 2009). With progress in technology and the advancement of biological material applications, the success rate of joint replacement surgery has continually increased. However, this surgery is often accompanied by complications, including aseptic prosthetic loosening and periprosthetic osteolysis (Bai et al., 2017; Brinkmann et al., 2012). Implanted joint replacement derived wear particles are believed to be the major inducer of local inflammation and periprosthetic bone loss, thus ultimately resulting in failure of the prostheses (Czaja, 2011). During this process, inflammatory cells (such as macrophages and lymphocytes) are recruited to the surface of the prosthesis and local bone tissues (Cody et al., 2011). These cells are activated to release proinflammatory cytokines (such as interleukin [IL]‐1, IL‐6, tumor necrosis factor alpha [TNF‐α], and PGE2), which promote the expression of RANKL, which, in turn, contributes to osteoclastogenesis (Castillo et al., 2012; Cuetara et al., 2006). Additionally, the binding of RANKL and RANK activates the mitogen‐activated protein kinase (MAPK) and nuclear factor‐κB (NF‐κB) signaling pathways (Feng, 2005; Goldring et al., 1983), which have been demonstrated to be crucial for osteoclast formation and bone absorption function. In summary, wear particle mediated osteoclastogenesis and inflammation are the key factors initiating osteolysis and aseptic loosening (Harris & is, 2001). Osteoclasts are differentiated from monocytes and macrophages, and have been demonstrated to be resorptive cells in bone metabolism (Liu et al., 2013). In osteoclastogenesis, hematopoietic osteoclast precursors differentiate into mature osteoclasts; this process is central to bone destruction and resorption (osteolysis).

Autophagy is traditionally identified as a dynamic catabolic process, in which the damaged proteins or organelles enter autophagosomes and are delivered to lysosomes for degradation (Levine & Kroemer, 2008; Li et al., 2014; Liao et al., 2012; Liu et al., 2015). Autophagy occurs when cells are exposed to adverse conditions, including nutrient deprivation, hypoxia, radiation, and pathogenic infection (Mizushima, 2007). During this process, increased expression of ATG proteins is the main contributor to the formation of double‐membrane autophagosome vesicles, which eventually fuse with lysosomes. Acid proteases and hydrolases in the autolysosomes degrade the engulfed autophagosomes. The digested products of autophagosomes, amino acids and fatty acids, are then transported into the cytoplasm to be used again for nutrition (Mediero et al., 2015; Mizushima & Levine, 2010). Studies increasingly indicate links between autophagy and various physiological processes and diseases. Beyond the previously described functions in homeostasis maintenance, autophagy is involved in the development and differentiation of various cell types, such as erythrocytes, lymphocytes, adipocytes, and macrophages (Mazière et al., 2009; Nakahira et al., 2011; Purdue et al., 2006).

Netrin‐1, a member of the axonal guidance protein family, is also involved in the differentiation and formation of osteoclasts. Antibody blockade of Netrin‐1 or Unc5b inhibits osteoclast differentiation (Purdue et al., 2007). Thus, Netrin‐1 is likely to play an important role in the osteoclastogenesis induced by wear particles and the resultant bone resorption. Although studies have shown that Netrin‐1 ultimately influences osteoclast differentiation through RhoA and FAK, the specific molecular signaling pathway mechanisms have not been clearly elucidated. Studies have shown that in a model of myocardial infarction, Netrin‐1 improves cardiac function through signaling by its receptors, DCC and ERK1/2, thus decreasing the occurrence of infarcts (Qin et al., 2011). In addition, Netrin‐1 increases the expression of the autophagy marker Beclin‐1 and the LC3‐II/LC3‐I ratio by increasing MAPK phosphorylation and decreasing mTOR phosphorylation (Razani et al., 2012). Other studies have shown that blockade of the NF‐κB and MAPK pathways attenuates wear particle‐induced osteoclast differentiation (Rawi et al., 2011). Together, these findings indicate that Netrin‐1 is closely associated with activation of the MAPK signaling pathway and cellular autophagy.

Although osteoclastogenesis plays an important role in bone destruction and resorption, the mechanisms of differentiation from osteoclast progenitor cells into osteoclasts has not yet been elucidated. The effects of autophagy on the osteoclastogenesis induced by wear particles also remain unclear. We speculate that Netrin‐1 promotes autophagy caused by wear particles and promotes the formation of osteoclasts through the ERK1/2 signaling pathway.

## 2 MATERIALS AND METHODS

### 2.1 Cell line and cell culture

RAW 264.7 mouse monocyte macrophages were obtained in our laboratory. After resuscitation, cells were cultured in Dulbecco's modified Eagle's medium containing 10% fetal bovine serum (Gibco, Life Technologies), 100 U/ml penicillin and 100 mg/ml streptomycin. The cells were incubated in a humidified atmosphere of 95% air and 5% CO_2_ at 37°C. Autophagic function was determined on the basis of GFP‐LC3 cleavage by using the adenovirus AdGFP‐LC3 (Cyagen Biosciences Inc.) to express a cleavable GFP‐LC3 fusion protein. Viruses were amplified in HEK‐293 cells, and purified twice on cesium chloride gradients, as previously described; the titer was determined with plaque assays (Rawi et al., 2011). At 70% confluence, RAW 264.7 cells were infected at an multiplicity of infection of 50 for 24 h; afterward, the infected cells were stimulated with nano‐scale titanium (Ti) wear particles in the presence or absence of 3‐MA for 48 h, and then cell culture supernatants were collected for enzyme‐linked immunosorbent assay (ELISA), protein and messenger RNA extraction and immunofluorescence staining. Ti particles with an average diameter of 30–45 nm were provided by the New Materials Research Institute of Xi'an Jiaotong University. Levels of free GFP cleaved from adenoviral GFP‐LC3 were observed by fluorescence microscopy.

### 2.2 Real time‐qPCR

The collected cells and implanted bones were collected for total RNA extraction with Trizol reagent (Invitrogen). The complementary DNAs were synthesized with reverse transcription kits (Quant One Step RT‐PCR kit; TIANGEN), and real‐time PCR was performed with SYBR green master mix (Quant one step qRT‐PCR Kit; TIANGEN) on a MyiQTM2 instrument (Bio‐Rad). β‐Actin was used to normalize the real‐time PCR data. The primer sequences are listed in Table 1.

**Table 1:** Primer sequences for quantitative reverse transcription PCR

Primer	Sequences
TRAP	5’‐TGACAAGAGGTTCCAGGA‐3’
	5’‐AGCCAGGACAGCTGAGTG‐3’
RANKL	5’‐GAAAGGCTTGTTTCATCCTCC‐3’
	5’‐GACTCCATGAAAACGCAGGT‐3’
OPG	5’‐GCAAGTACTTTGGAGGAAGGG‐3’
	5’‐CCACAATGAACAAGTGGCTG‐3’
IL‐1β	5’‐GGTCAAAGGTTTGGAAGCAG‐3’
	5’‐TGTGAAATGCCACCTTTTGA‐3’
IL‐6	5’‐ACCAGAGGAAATTTTCAATAGGC‐3’
	5’‐TGATGCACTTGCAGAAAACA‐3’
TNF‐α	5’‐AGGGTCTGGGCCATAGAACT‐3’
	5’‐CCACCACGCTCTTCTGTCTAC‐3’
β‐actin	5’‐ATGGAGGGGAATACAGCCC‐3’
	5’‐TTCTTTGCAGCTCCTTCGTT‐3’
Atg5	5’‐GCAACTCTGGATGGGATTGC‐3’
	5’‐CAACTGTCCATCTGCAGCCA‐3’
Beclin1	5’‐CTCCCGAGGTGAAGAGCATC‐3’
	5’‐TTCCTCCTGGGTCTCTCCTG‐3’
Atg7	5’‐CGGCGGATCCAATTCCTGTA‐3’
	5’‐GGATGCACTGGATACCAGCA‐3’
Atg12	5’‐AAGTGGGCAGTAGAGCGAAC‐3’
	5’‐CACGCCTGAGACTTGCAGTA‐3’
Netrin‐1	5’‐CCCTGGAGAAATGACGAGACG‐3’
	5’‐CAGTTCTCCTCCCGTCCAC‐3’

Abbreviations: IL, interleukin; TNF, tumor necrosis factor.

### 2.3 Western blotting

The cells and pouch wall tissues were lysed in RIPA buffer supplemented with protease inhibitor cocktail (Roche) to prepare the protein samples. Protein concentrations were measured with an Enhanced Bicinchoninic Acid Protein Assay Kit (Beyotime Biotechnology), and 40 μg of protein was loaded and separated with 10% sodium dodecyl sulfate polyacrylamide gel electrophoresis. Proteins were transferred to polyvinylidene difluoride (PVDF) membranes and then blocked with 5% bovine serum albumin in Tris‐buffered saline with Tween 20 (TBST) before immunodetection with antibodies to the following proteins: LC‐3 (1:1000; Abcam), TRAP (1:1000; Abcam), Beclin1 (1:1000; Abcam), Netrin‐1 (1:1000; Abcam), RANKL (1:1000; Abcam), OPG (1:1000; Abcam), ERK (1:1000; CST), p‐ERK (1:1000; CST), β‐actin (1:1000, CST), Unc5b (200 µg/ml, Abcam), DCC (200 µg/ml; Abcam) and recombinant Netrin‐1 (250 ng/ml; R&D). After 16 h of primary antibody incubation, the PVDF membranes were washed with TBST before being incubated with secondary antibody for 1.5 h. Specific binding was visualized by ECL reaction (生产商). The western blot bands were quantified in ImageJ Software (version 1.41).

### 2.4 Immunohistochemical and immunofluorescence staining

Cells and implanted bones were fixed with 4% paraformaldehyde, then incubated with primary antibody against TRAP; this was followed by washing and incubation with FITC‐conjugated goat anti‐rabbit secondary antibodies (1:250; Earthox) for 2 h at 30°C. The nuclei were stained with 4’,6‐diamidino‐2’‐phenylindole. Fluorescence images were visualized and captured with an inverted fluorescence microscope (Olympus). The bones were embedded in paraffin and sliced into 4‐μm sections, and this was followed by dewaxing and rehydration. The sections were incubated with primary antibody against TRAP, then washed and incubated with horseradish peroxidase‐conjugated goat anti‐rabbit secondary antibody (1:250; Earthox) for 2 h at room temperature. Images were visualized and captured with a phase contrast microscope (Olympus).

### 2.5 Establishment of the bone resorption air pouch model and naringin intervention

The animal experiments were performed in accordance with the guidelines of the Chinese Council on Animal Care, and the study was approved by the Xi'an Jiaotong University Committee (Xi'an, China). The air pouch model of bone resorption was established in our laboratories as described previously (Ravikumar et al., 2010; Ru et al., 2016). Briefly, air pouches were generated by injection of sterile air into the backs of female BALB/c mice (8 weeks of age). At Day 7 after air pouch formation, a proximal or distal section of the femur, or a section of calvarium from congeneric littermate donors, was surgically implanted into the established air pouches. Ti particles with or without 3‐MA were injected into each pouch, and the mice were killed 7 days later to harvest implanted bones, pouch wall tissues and blood for further measurements.

### 2.6 Enzyme‐linked immunosorbent assay

Cell culture supernatants and serum were analyzed with a Mouse RANKL ELISA Kit (Abcam) and Mouse Osteoprotegerin ELISA Kit (Abcam). All experiments were performed according to the manufacturer's protocols.

### 2.7 Pit resorption assay

After the coculture, the lower chamber bone ground sections were removed, washed with phosphate buffered saline three times and treated with 2.5% ethylenediaminetetraacetic acid and trypsin for 10 min to digest the cells on the bone ground sections. After fixation with 2.5% glutaraldehyde for 30 min, the impurities in the bone resorption pits were removed by ultrasonic washing. Then, alcohol gradient dehydration (30%–100% ethanol solution) was performed, and finally isoamyl acetate was added to replace the 100% ethanol. After vacuum drying, the bone resorption pits were observed under a scanning electron microscope.

### 2.8 Transmission electron microscopy

The bone pieces were quickly cut into sections approximately 1 mm thick and fixed with 2.5% glutaraldehyde at room temperature. After fixation, sections were rinsed with 0.2 M phosphate buffer (pH 7.2). Then the samples were treated with 1% buffered osmium tetroxide for 1 h, dehydrated in gradient ethanol and embedded in epoxy resin to prepare ultrathin sections, mounted on a copper grid, and stained with acetate and lead acetate with uranyl. Images were collected with a transmission electron microscope.

### 2.9 Statistical analysis

Data are presented as means ± *SEM* for each experiment. Statistical comparisons were performed as appropriate, with either Student's two‐tailed *t* test or analysis of variancewith Tukey's multiple comparison posttest. A *p* value less than .05 was considered significant.

## 3 RESULTS

### 3.1 Wear particles induce osteoclast differentiation by increasing TRAP expression

**Figure 1:**
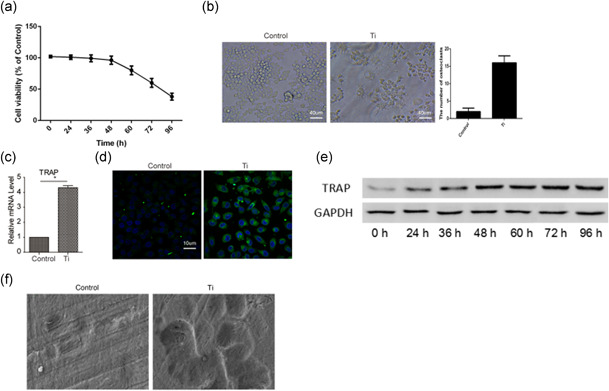
Wear particles induce osteoclast differentiation, on the basis of increased expression of TRAP. (a) The cell viability of RAW 264.7 cells was detected with CCK‐8 assays after treatment with Ti wear particles. (b) Phase contrast microscopic images of RAW 264.7 cells with or without treatment with Ti wear particles. (c) The mRNA expression of TRAP in RAW 264.7 cells with or without treatment with Ti wear particles. (d) The immunofluorescence staining of TRAP in RAW 264.7 cells with or without treatment with Ti wear particles. (e) Western blot detection of TARP expression in RAW 264.7 cells with or without treatment with Ti wear particles. (f) Pit resorption assay indicating the effect of Ti treatment on osteoclast differentiation. Data represent mean ± *SEM; n* = 5 (*p < .05). mRNA, messenger RNA

To investigate the effect of wear particles on osteoclastogenesis in vitro, we cultured RAW 264.7 cells and exposed them to wear debris. We first observed cell viability and morphology changes. The decrease in cell viability was proportionate to the incubation time (Figure 1a). Ti treatment induced more cell projections and cell fusion (Figure 1b). The expression of TRAP (an osteoclast marker) was then detected by real time‐qPCR, immunofluorescence staining and western blotting. Ti treatment upregulated the expression of TRAP in macrophages (Figure 1c–e). Pit resorption assays also demonstrated the effects of Ti treatment on osteoclast differentiation (Figure 1f). Together, the results indicated that wear particles induce osteoclast differentiation.

### 3.2 Wear particles induce autophagy in macrophages

**Figure 2:**
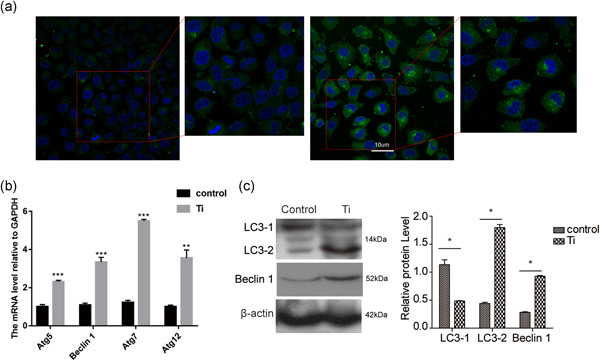
Wear particles induce autophagy of macrophages. (a) RAW 264.7 cells were transfected with GFP‐LC3 for 24 h and then treated with Ti for 48 h to observe the punctuate GFP‐LC3 distribution by fluorescence microscopy. (b) The mRNA expression of Atg5, Beclin1, Atg7, and Atg12 in RAW 264.7 cells with or without treatment with Ti wear particles. (c) The expression of LC‐3 was detected by western blotting in RAW 264.7 cells with or without treatment with Ti wear particles. Data represent mean ± *SEM; n* = 5 (*p < .05). mRNA, messenger RNA

The formation of autophagosomes from a cup‐shaped single membrane structure is the major event in autophagy, which is characterized by the conversion of the microtubule‐associated protein 1 light chain 3 (LC3) from a diffuse cytosolic form (LC3‐I) to a lapidated form (LC3‐II). SoLC3 and other ATG family proteins are typically used as markers of autophagy. In our study, RAW 264.7 cells were transfected with GFP‐LC3 for 24 h and then treated with Ti for 48 h, and changes in GFP‐LC3 localization were observed. Ti clearly induced a punctuate GFP‐LC3 (Figure 2a). Treatment with Ti promoted the expression of Atg5, Atg7, Atg12, and LC3‐II (Figure 2b,c). Additionally, we detected another protein, Beclin‐1, an autophagy activator. As expected, the expression of Beclin‐1 was also clearly higher in Ti treated cells than in untreated cells, on the basis of real time‐qPCR and western blotting (Figure 2b,c). These results demonstrated that wear particles induce autophagy in macrophages.

### 3.3 Wear particles regulate autophagy by increasing the expression of Netrin‐1

**Figure 3:**
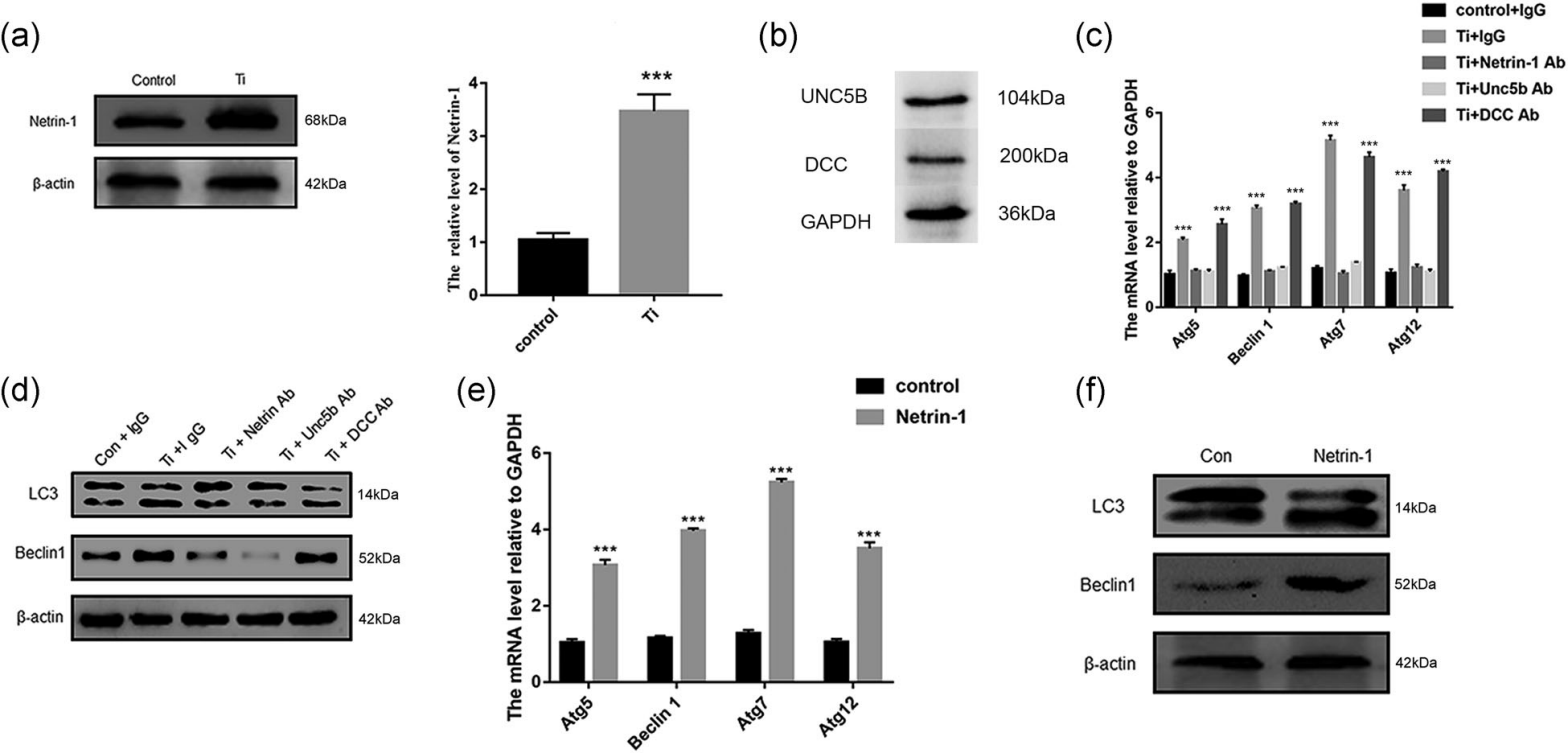
Wear particles regulate autophagy by increasing the expression of Netrin‐1. (a) Western blot detection of Netrin‐1 expression in RAW 264.7 cells with or without treatment with Ti wear particles. (b) The mRNA expression of Netrin‐1 in RAW 264.7 cells with or without treatment with Ti wear particles. (c) The protein expression of UNC5b and DCC in RAW 264.7 cells. (d) The mRNA expression of Atg5, Beclin1, Atg7, and Atg12 in RAW 264.7 cells with or without treatment with Ti wear particles and IgG, Netrin‐1, Unc5b, or DCC neutralizing antibodies. (e) The expression of LC‐3 and Beclin1 was detected by western blotting in RAW 264.7 cells with or without treatment with Ti wear particles and IgG, Netrin‐1, Unc5b, or DCC neutralizing antibodies. (f) The mRNA expression of Atg5, Beclin1, Atg7, and Atg12 in RAW 264.7 cells with or without treatment with recombinant Netrin‐1. (g) The expression of LC‐3 and Beclin1 was detected by western blotting in RAW 264.7 cells with or without treatment with recombinant Netrin‐1. mRNA, messenger RNA

We investigated the role of Netrin‐1 in Ti‐induced macrophage autophagy models. Ti treatment promoted the expression of Netrin‐1 in RAW 264.7 cells (Figure 3a,b). UNC5b and DCC were detected in RAW 264.7 cells (Figure 3c). Further studies demonstrated that neutralizing antibodies against Netrin‐1 and its receptor Unc5b effectively decreased the Atg5, Atg7, Atg12, Beclin‐1, and LC3‐II expression was induced by Ti, whereas neutralizing antibodies to DCC, another receptor of Netrin‐1, did not have this effect (Figure 3d,e). After treating the cells with recombinant Netrin‐1, we observed an increase in Atg5, Atg7, Atg12, Beclin‐1, and LC3‐II (Figure 3f,g). These results indicated that Ti induced autophagy was mediated by Netrin‐1 and its receptor Unc5b. Netrin‐1 thus plays an important role in the development of autophagy in macrophages.

### 3.4 Blocking of autophagy inhibits wear particle induced osteoclastogenesis in vitro

**Figure 4:**
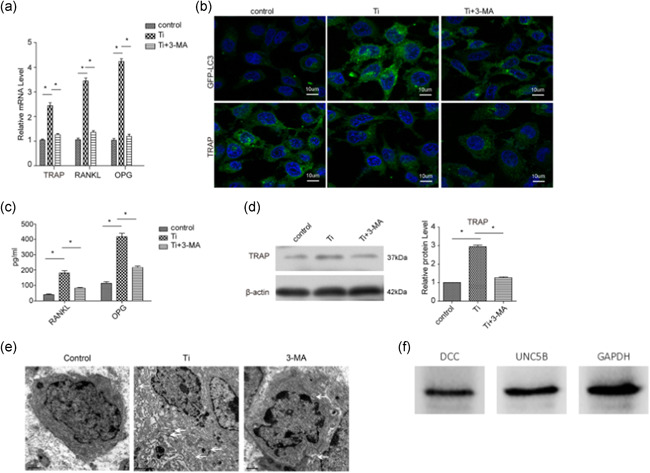
Blocking of autophagy inhibits wear particle induced osteoclastogenesis in vitro. (a) The expression of TRAP, RANKL, and OPG was detected by real‐time PCR in RAW 264.7 cells under the indicated treatments. (b) RAW 264.7 cells were transfected with GFP‐LC3 for 24 h and then treated with Ti in the presence or absence of 3‐MA for 48 h to observe the punctuate GFP‐LC3 distribution by fluorescence microscopy; the expression of TRAP was also detected by immunofluorescence staining. (c) The cell culture supernatants in different groups were collected to detect RANKL and OPG with ELISA. (d) The expression of TRAP in RAW 264.7 cells in different groups was detected by western blotting. (e) Ti or 3‐MA treatment induced autophagy was detected by electron microscopy of bone sections. Data represent mean ± *SEM; n* = 5 (*p < .05). (f) The expression of DCC and UNC5B were tested by the western blotting. ELISA, enzyme‐linked immunosorbent assay; mRNA, messenger RNA

We next investigated the effect of autophagy on osteoclast development by suppressing the formation of autophagosomes. The expression of TRAP was upregulated when RAW 264.7 cells were exposed to Ti wear particles; however, the TRAP expression was decreased by the addition of 3‐MA (Figures 4a,b, and 4d). Previous research has identified that RANKL and OPG play important roles in the differentiation from osteoclast precursor cells to osteoclasts. Therefore, we also detected the expression of RANKL and OPG during osteoclastogenesis induced by wear particles. In agreement with the results above, the expression of RANKL and OPG also clearly increased after stimulation with Ti wear particles; however, the increase was inhibited with 3‐MA treatment (Figures 4a and 4c). We also found that autophagy was induced by Ti but was reversed by addition of 3‐MA, on the basis of electron microscopy of bone sections, (Figure 4e). The expression of DCC and UNC5B were tested by the western blotting (Figure 4f). Together, these findings demonstrated that Ti not only induces osteoclastogenesis by enhancing the expression of TRAP but also upregulates the expression of RANKL and OPG.

### 3.5 Netrin‐1 significantly promotes osteoclastogenesis

**Figure 5:**
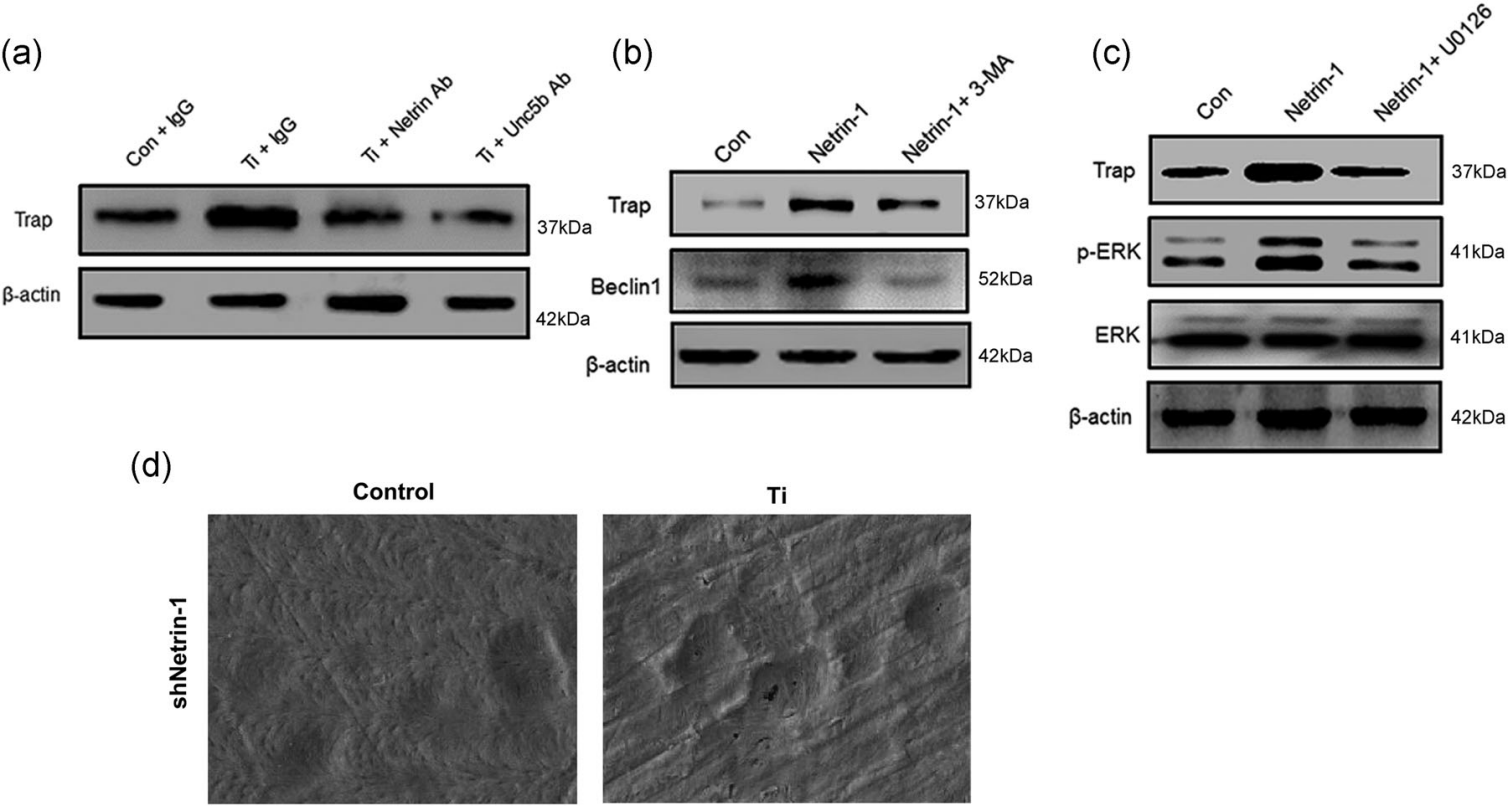
(a) Netrin‐1 significantly promotes osteoclastogenesis. The expression of Trap was detected by western blotting in RAW 264.7 cells with or without treatment with Ti wear particles and IgG, Netrin‐1, or Unc5b neutralizing antibodies. (b) The expression of Trap and Beclin1 was detected by western blotting in RAW 264.7 cells with or without treatment with recombinant Netrin‐1 and 3‐MA. (c) The expression of Trap, p‐ERK and ERK was detected by western blotting in RAW 264.7 cells with or without treatment with recombinant Netrin‐1 and U0126. (d) Pit resorption assay for the effect of Netrin‐1 on osteoclast differentiation. IgG, immunoglobulin G

After having observed the effects of Netrin‐1 on autophagy of macrophages, we further examined its functions in osteoclastogenesis. The results indicated that TRAP was upregulated when RAW 264.7 cells were exposed to Ti wear particles; however, the TRAP expression was decreased with the addition of Netrin‐1 neutralizing antibodies and Unc5b (Figure 5a). Furthermore, the expression of TRAP and Beclin‐1 was upregulated when RAW 264.7 cells were treated with Netrin‐1; however, these effects, particularly Beclin‐1 expression, were reversed by 3‐MA treatment (Figure 5b). For further elucidation, we explored the roles of ERK1/2 signaling in osteoclastogenesis. The results indicated that the expression of TRAP and p‐ERK was upregulated when Netrin‐1 was added to RAW 264.7 cells, and these effects were inhibited by U0126, a p‐ERK inhibitor (Figure 5c). Pit resorption assays also demonstrated the role of Netrin‐1 in osteoclast differentiation (Figure 5d). In addition to influencing macrophage autophagy, Netrin‐1 affects osteoclast formation through the ERK1/2 signaling pathway.

### 3.6 Blocking of autophagy inhibits wear particle induced osteoclastogenesis in vivo

**Figure 6:**
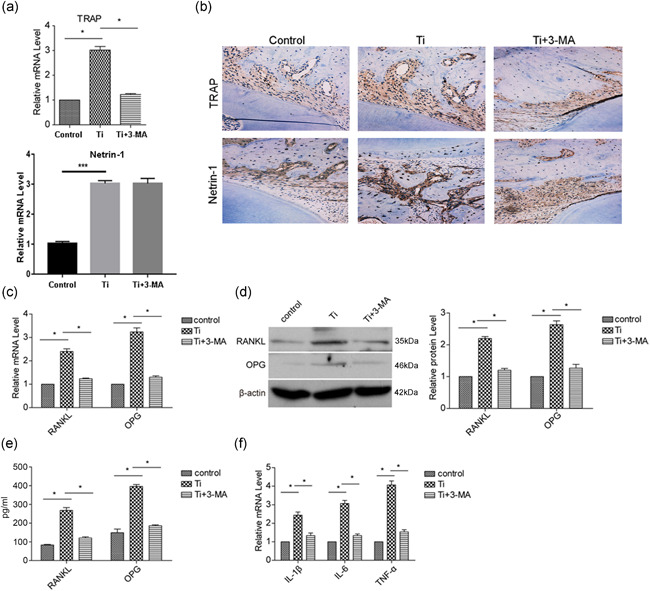
Blocking of autophagy inhibits wear particle induced osteoclastogenesis in vivo. The air pouch mold was established, and Ti wear particles with or without 3‐MA were injected in the air pouch. (a) After 7 days, the implanted bones were harvested to detect the mRNA expression of TRAP and Netrin‐1 with real‐time PCR. (b) The harvested bones were also subjected to immunohistochemical staining to detect the expression of TRAP and Netrin‐1. (c) The mRNA expression of RANKL and OPG in pouch wall tissues was detected by real‐time PCR. (d) The protein expression of RANKL and OPG in pouch wall tissues was detected by western blotting. (e) The serum levels of RANKL and OPG were detected by ELISA. The mRNA expression of proinflammatory factors (IL‐1β, IL‐6, and TNF‐α) in pouch wall tissues was measured to assess the degree of inflammation. Data represent mean ± *SEM; n* = 5 (*p < .05). ELISA, enzyme‐linked immunosorbent assay; IL, interleukin; mRNA, messenger RNA; TNF, tumor necrosis factor

Next, we established an air pouch model to investigate the role of autophagy in osteoclast development. the expression of TRAP and Netrin‐1 increased in implanted bone in response to Ti wear particle treatment, whereas administration of 3‐MA weakened the expression of TRAP but not Netrin‐1 (Figure 6a,b). In pouch wall tissues, Ti wear particles enhanced RANKL and OPG expression, and this effect was reversed by administration of 3‐MA (Figures 6c,d and 6e). Moreover, we measured the proinflammatory factors IL‐1β, IL‐6, and TNF‐α in pouch wall tissues to assess the degree of inflammation due to different treatments. Ti wear particles induced clear inflammation in the bone implanted microenvironment, but this effect was weakened by 3‐MA treatment (Figure 6f). These data demonstrated that Ti wear particles induce osteoclastogenesis by upregulating TRAP, RANKL, and OPG, which may facilitate differentiation of progenitor cells into osteoclasts in vivo, in a manner controlled by autophagy. Simultaneously, Netrin‐1 expression induced by Ti wear particles was not affected by autophagy, In addition, Netrin‐1 promoted autophagy and osteoclastogenesis through the ERK1/2 signaling pathway. Ti wear particle induced inflammation was additionally found to be regulated by autophagy.

## 4 DISCUSSION

Under normal physiological conditions, autophagy can clear damaged organelles and insoluble protein aggregates in cells, and contribute to cell homeostasis maintenance and cell survival regulation (Rosenfeldt & Ryan, 2009; Rubinsztein et al., 2012). Autophagy occurs when cells are challenged, such as under starvation or pathogen infection. Previous studies have confirmed that autophagy plays an important role in intracellular pathogen clearance, antigen presentation and lymphocyte differentiation (Ren et al., 2004). Roles of autophagy in macrophages, including oxidative and inflammatory stress suppression, and polarization control, have also been reported (Sakiyama et al., 2001; Saitoh et al., 2008; Sato & Sato, 2011; Shi et al., 2012; Shanbhag et al., 1995; Shintani & Klionsky, 2004; Thomas et al., 2012; Tsukamoto et al., 2008).

Artificial joint replacement surgery is an important treatment for arthropathy; however, complications such as septic prosthetic loosening and periprosthetic osteolysis occur very often after this surgery. Wear particle derived implanted joint replacements have been confirmed to initiate local inflammation, periprosthetic bone loss and even the ultimate failure of prostheses. The aim of our study was to investigate the effect of Netrin‐1 and autophagy on osteoclast development in vitro and vivo.

The expression profile of the RAW 264.7 cell line is close to that of osteoclasts (Todde et al., 2009); consequently, this cell line has frequently been used in in vitro models to investigate osteoclast differentiation precursors in response to wear debris (Tsao et al., 2008; Udagawa et al., 1990; Wooley & Schwarz, 2004; Xiao et al., 2015). As reported, we observed the presence TRAP expression and multiple nuclei—the characteristic features of mature osteoclasts (Yang et al., 2012). We detected the expression of TRAP in RAW 264.7 cells with Ti wear particle treatment. A mouse model based study has used 30 mg of Ti particles embedded under the periosteum at the middle suture of the calvaria, with a Ti particle concentration of 0.1 mg/ml (Zhao et al., 2012). Such particles have been shown to effectively mimic the wear particles retrieved from periprosthetic tissues (Zhou et al., 2011). Nano‐sized Ti particles were used in this study at a concentration of approximately 0.0012 mg/ml. The ratio of 100 particles/cell used in the experiment to the ratio of 0.1 mg/ml microparticles delivered to cells was consistent.

As expected, treatment with Ti wear particles enhanced the expression of TRAP and resulted in formation of more cell projections and multiple cell fusion, thus indicating that Ti wear particles promote osteoclast development. Moreover, treatment with Ti wear particles also caused increased expression of Netrin‐1. Studies have shown that Netrin‐1 participates in differentiation and osteoclastogenesis through its receptor Unc5b. The Netrin‐1 neutralization experiment in our study was consistent with the previous findings. Further experiments also revealed that the ERK1/2 signaling pathway mediates this process. Autophagosome formation is accompanied by LC3 conversion from LC3‐I to LC3‐II. This process is regarded as a marker of autophagy. To investigate the effect of autophagy on the osteoclastogenesis induced by wear particles, we transfected RAW 264.7 cells with GFP‐LC3 before treatment and observed a clear punctuate GFP‐LC3 distribution in macrophages. We also found that LC3‐II protein increased in Ti wear particle treated RAW 264.7 cells. These results demonstrated that autophagy was activated during the osteoclast differentiation. Next, we further investigated whether autophagy might affect osteoclast differentiation induced by wear particles. According to previous reports, 3‐MA inhibits the formation of autophagosomes, thereby blocking autophagy flux. As shown in our results, 3‐MA treatment decreased the expression of TRAP, thus indicating that autophagy deficiency suppress osteoclastogenesis. However, 3‐MA treatment did not prevent the increase in Netrin‐1 expression. These results indicated that has Netrin‐1 is not regulated by autophagy. Netrin‐1 facilitates autophagy and osteoclastogenesis. In addition, we found that Ti wear particles induced expression of RANKL and OPG, both of which have been demonstrated to promote differentiation from macrophages to osteoclasts. OPG and RANKL are generally produced by osteoblast cell lineages. However, we detected their expression in RAW 264.7 cells, in agreement with a previous report. We speculate that wear particles may mediate the expression of OPG and RANKL in RAW 264.7 cells, although the specific mechanism must be further investigated. Moreover, we found that both RANKL and OPG increased. OPG, a decoy receptor, competitively binds RANKL and prevents the osteoclast formation. After the addition of wear particles, RANKL increased, and OPG decreased in RAW 264.7 cells. As a result of cell self‐regulation, the increase in OPG negatively regulated the overactivation of RANKL, and at the time points in our experiment, no reduction in OPG was detected. A previous study supports these findings. Although the exact mechanisms require further exploration, 3‐MA treatment inhibited the increase in RANKL and OPG induced by Ti wear particles, thereby suggesting that autophagy contributes to the development and maturation of osteoclasts caused by wear particles, in a manner at least partially dependent on the induction of RANKL and OPG. Furthermore, an air pouch model was established and used to explore autophagy functions in osteoclast formation. The results from in vivo experiments also supported the conclusions based on in vivo experiments. Autophagy accelerated the osteoclastogenesis induced by wear particles, and blocking autophagy also inhibited osteoclast maturation. In addition, proinflammatory factors (IL‐1β, IL‐6, and TNF‐α) decreased with 3‐MA treatment, thereby indicating that autophagy caused by wear particles not only affects osteoclast development but also regulates inflammation. However, these proinflammatory factors are released by many immune cells. We have not identified which cell type is the major source and how autophagy affects the expression of proinflammatory factors.

In conclusion, our study reveals that disruption of autophagy arrests the differentiation of macrophages into osteoclasts and ameliorates the inflammation induced by wear particles. These data suggest that autophagy may be a novel protective cellular response during osteolysis induced by wear particles. However, the molecular mechanisms linking autophagy and inflammation induced by wear particles remain to be investigated.

## CONFLICT OF INTERESTS

The authors declare that there are no conflict of interests.

## Data Availability

The authors agree with Expects Data Policy.
